# Computing linkage disequilibrium aware genome embeddings using autoencoders

**DOI:** 10.1093/bioinformatics/btae326

**Published:** 2024-05-22

**Authors:** Gizem Taş, Timo Westerdijk, Eric Postma, Project MinE ALS GWAS Consortium, Wouter van Rheenen, Mark K Bakker, Kristel R van Eijk, Maarten Kooyman, Ahmad Al Khleifat, Alfredo Iacoangeli, Nicola Ticozzi, Johnathan Cooper-Knock, Marta Gromicho, Siddharthan Chandran, Karen E Morrison, Pamela J Shaw, John Hardy, Michael Sendtner, Thomas Meyer, Nazli Başak, Isabella Fogh, Adriano Chiò, Andrea Calvo, Elisabetta Pupillo, Giancarlo Logroscino, Marc Gotkine, Patrick Vourc’h, Philippe Corcia, Philippe Couratier, Stèphanie Millecamps, François Salachas, Jesus S Mora Pardina, Ricardo Rojas-García, Patrick Dion, Jay P Ross, Albert C Ludolph, Jochen H Weishaupt, Axel Freischmidt, Gilbert Bensimon, Lukas Tittmann, Wolfgang Lieb, Andre Franke, Stephan Ripke, David C Whiteman, Catherine M Olsen, Andre G Uitterlinden, Albert Hofman, Philippe Amouyel, Bryan Traynor, Adrew B Singleton, Miguel Mitne Neto, Ruben J Cauchi, Roel A Ophoff, Vivianna M van Deerlin, Julian Grosskreutz, Caroline Graff, Lev Brylev, Boris Rogelj, Blaž Koritnik, Janez Zidar, Zorica Stević, Vivian Drory, Monica Povedano, Ian P Blair, Matthew C Kiernan, Garth A Nicholson, Anjali K Henders, Mamede de Carvalho, Susana Pinto, Susanne Petri, Markus Weber, Guy A Rouleau, Vincenzo Silani, Jonathan Glass, Robert H Brown, John E Landers, Christopher E Shaw, Peter M Andersen, Fleur C Garton, Allan F McRae, Russell L McLaughlin, Orla Hardiman, Kevin P Kenna, Naomi R Wray, Ammar Al-Chalabi, Philip Van Damme, Leonard H van den Berg, Jan H Veldink, Jan H Veldink, Alexander Schönhuth, Marleen Balvert

**Affiliations:** Department of Econometrics and Operations Research, Tilburg University, Tilburg 5037AB, The Netherlands; Department of Neurology, University Medical Center Utrecht, Utrecht 3584CX, The Netherlands; Department of Cognitive Science and Artificial Intelligence, Tilburg University, Tilburg 5037AB, The Netherlands; Department of Neurology, University Medical Center Utrecht, Utrecht 3584CX, The Netherlands; Faculty of Technology, Bielefeld University, Bielefeld 33615, Germany; Department of Econometrics and Operations Research, Tilburg University, Tilburg 5037AB, The Netherlands

## Abstract

**Motivation:**

The completion of the genome has paved the way for genome-wide association studies (GWAS), which explained certain proportions of heritability. GWAS are not optimally suited to detect non-linear effects in disease risk, possibly hidden in non-additive interactions (epistasis). Alternative methods for epistasis detection using, e.g. deep neural networks (DNNs) are currently under active development. However, DNNs are constrained by finite computational resources, which can be rapidly depleted due to increasing complexity with the sheer size of the genome. Besides, the *curse of dimensionality* complicates the task of capturing meaningful genetic patterns for DNNs; therefore necessitates dimensionality reduction.

**Results:**

We propose a method to compress single nucleotide polymorphism (SNP) data, while leveraging the linkage disequilibrium (LD) structure and preserving potential epistasis. This method involves clustering correlated SNPs into haplotype blocks and training per-block autoencoders to learn a compressed representation of the block’s genetic content. We provide an adjustable autoencoder design to accommodate diverse blocks and bypass extensive hyperparameter tuning. We applied this method to genotyping data from Project MinE, and achieved 99% average test reconstruction accuracy—i.e. minimal information loss—while compressing the input to nearly 10% of the original size. We demonstrate that haplotype-block based autoencoders outperform linear Principal Component Analysis (PCA) by approximately 3% chromosome-wide accuracy of reconstructed variants. To the extent of our knowledge, our approach is the first to simultaneously leverage haplotype structure and DNNs for dimensionality reduction of genetic data.

**Availability and implementation:**

Data are available for academic use through Project MinE at https://www.projectmine.com/research/data-sharing/, contingent upon terms and requirements specified by the source studies. Code is available at https://github.com/gizem-tas/haploblock-autoencoders.

## 1 Introduction

The complete sequencing of the human genome enabled genome-wide association studies (GWAS) to seek relations between disease status and a vast number, often exceeding a million, of genetic variants (Hardy and Singleton 2009). The prospect of identifying the genetic factors contributing to variations in disease susceptibility holds the promise of developing treatments for currently incurable diseases (Manolio *et al.* 2009). Usually, the phenotypic variance explained by genetic variants falls behind its expected portion to be explained, by a difference known as the *missing heritability* (Maher 2008, Manolio *et al.* 2009). Taking amyotrophic lateral sclerosis (ALS) as an example, heritability is estimated around 45% (Ryan *et al.* 2019), which is only partially attributed to the latest GWAS-identified loci (van Rheenen *et al.* 2021). In case of many complex diseases, part of the heritability may be disguised in smaller effects of non-significant loci (Shi *et al.* 2016, Boyle *et al.* 2017). Furthermore, non-additive interactions (i.e. epistasis) between already identified loci might account for part of the missing heritability (Zuk *et al.* 2012, Blanco-Gómez *et al.* 2016). All in all, solving the mysterious genetics of complex diseases seems only likely when joint analysis of many variants, ideally the entire genome, is feasible.

The increasing availability of large-scale genotyping data has boosted interest in scalable deep neural networks (DNNs) (Wainberg *et al.* 2018). However, a DNN’s performance is limited by computational resources, which could easily be exhausted due to the exponential increase in network complexity given the sheer size of genotyping data. Genetic variants can amount to millions, typically much more than the samples due to practical, technical, and cost-related limitations (Gazestani and Lewis 2019). Processing large genetic input in its raw state would be computationally too heavy, even for a shallow network with one hidden layer, let alone a deeper one with several layers.

The challenge, known as the *curse of dimensionality*, can manifest itself in issues beyond computational complexity (Donoho 2000). For example, single nucleotide polymorphism (SNP) data can be sparse due to rare variants. Data sparsity can jeopardize generalization of learning models because of a high proportion of zeros, such that the model’s predictive ability suffers from overfitting (Altman and Krzywinski 2018). Or, the model could overlook the predictive power of sparse features and prioritize denser ones. The *curse of high dimensionality* thus complicates finding meaningful patterns for DNNs and may induce spurious genotype-phenotype associations.

One could rely on biological priors to alleviate this *curse*. The phenomenon of alleles at different loci being associated in a non-random manner is termed *Linkage Disequilibrium* (*LD*) (Slatkin 2008). High or complete LD between SNP pairs is often pointed out as the source of redundancy in large-scale genomic data and removing loci based on their pairwise correlation is a prevalent practice called LD pruning (Calus and Vandenplas 2018). From a practical standpoint, adopting a lower correlation threshold in such pre-selection methods would be advantageous in sweeping away more SNPs not only from high or complete LD pairs but also from moderately correlated ones. Therefore, relying solely on LD pruning to achieve the desired input dimensionality must be a rigorous reduction step, which could potentially impact future detection of epistatic interactions.

By quantifying the correlation between SNPs, LD can be used to segment genome-wide information into disjoint substructures. It is not unprecedented to divide the genome prior to dimensionality reduction, as a refuge from multi-collinearity. Hibar et al. summarized correlated SNPs within each gene into principal components that explain 95% of the variance per gene (Hibar *et al.* 2011). Li et al. proposed a local window approach, scanning each chromosome to form high-LD SNP clusters, which are then projected onto their principal components (Li *et al.* 2018). Despite its usefulness in disposing of the redundancy in sparse data, Principal Component Analysis (PCA) is only able to preserve variance as long as the SNPs can be assumed to interact linearly (McVean 2009, Alanis-Lobato *et al.* 2015). This would not be the ideal way out of the dimensionality issue when missing heritability is concerned, for bearing the risk of obscuring epistasis.

Autoencoders, on the other hand, offer a compelling solution due to their ability to compress high-dimensional genotype data effectively while preserving complex and non-linear patterns in the data (Bank *et al.* 2020). First designed as neural networks trained to reconstruct their input in the output layer (Rumelhart *et al.* 1986), autoencoders could learn abstract data representations, hence they were advertised as more powerful yet costly non-linear generalizations of PCA transformation (Hinton and Salakhutdinov 2006, Fournier and Aloise 2019). These concurrent utilities helped diversify their use cases, prominently in dimensionality reduction (Goodfellow *et al.* 2016).

**Figure 1.**
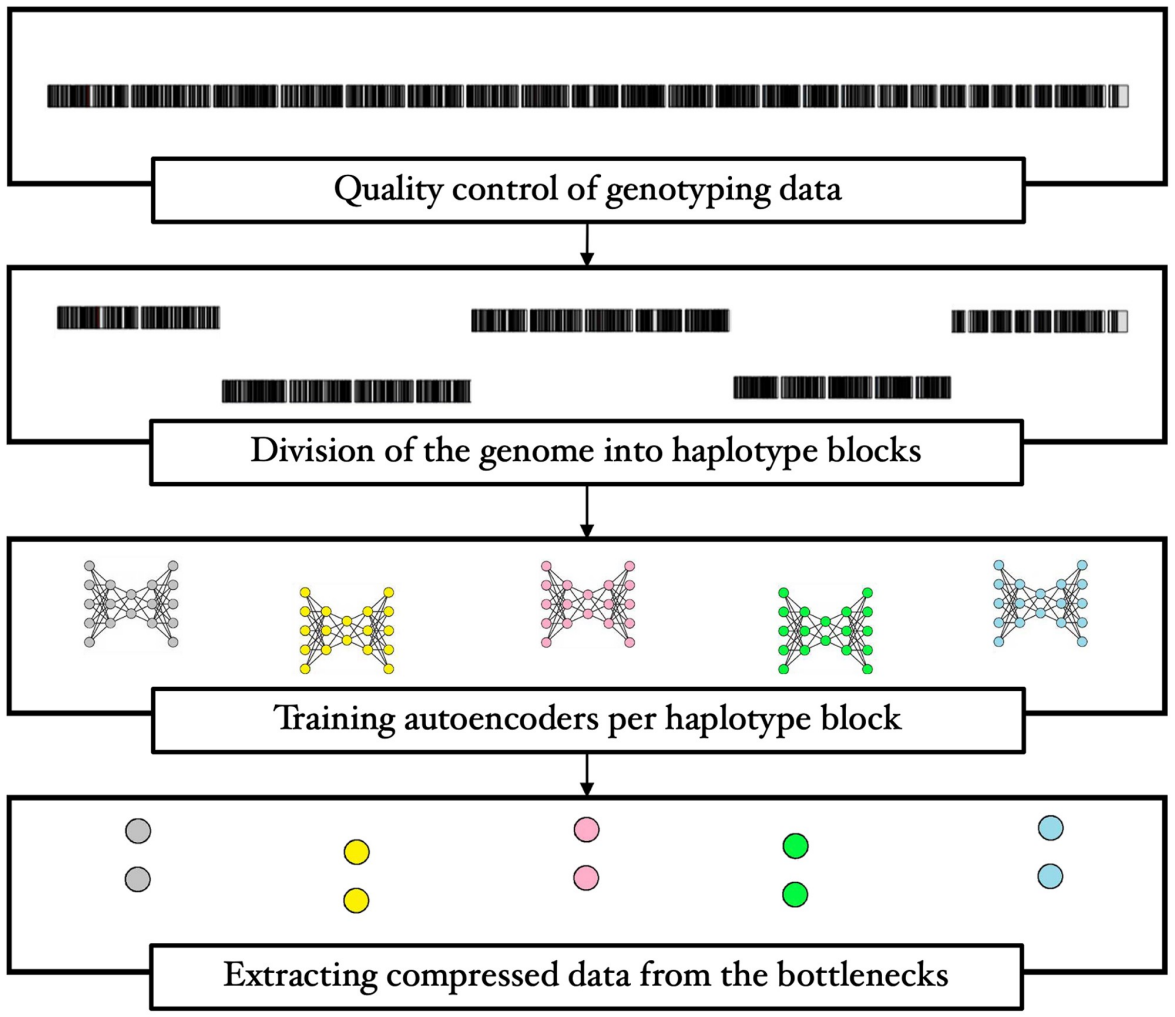
An overview of the workflow.

Above all, our aim is to compress high-dimensional genotype data in a way that preserves genetic patterns. Considering the uncharted genetic nature of complex diseases, we ought to take the quest further into scalability and non-linearity. We strive to find a balance between the *curse of dimensionality* and unwanted loss of information. Our strategy is to leverage inter-SNP correlations (LD), so as to maximize compression across disjoint genome segments. We hereby make use of haplotype blocks, hereinafter referred to as *haploblocks*, i.e. sections of the genome that have high internal LD (Barrett *et al.* 2005), to draw the boundaries between clusters of correlated SNPs. Next in our workflow (Fig. 1) comes the compression of each haploblock in a low-dimensional space through autoencoders. We hypothesize that exploiting the local correlation among SNPs could secure dual benefits, as it would not only mitigate the loss of valuable information but also overcome obstacles posed by the *curse of dimensionality*.

To achieve the proposed compression, many haploblocks need to be compressed and many autoencoders need to be trained. Given the variety of these blocks, optimization of each network’s hyperparameters would consume time and resources beyond practicality, even feasibility. Therefore, we seek a standardized way to build autoencoders, which manages the trade-off between compression rate and reconstruction accuracy. We investigate the link between certain characteristics of haploblocks and their corresponding optimal autoencoder configurations. Furthermore, we discuss the opportunities for favouring computational efficiency over marginal returns in performance. Following a versatile analysis, we finalize by compressing an entire chromosome and assess the performance of our method in terms of both quantified reduction and conservation of significant data patterns.

Our contribution is therefore 3-fold. First, we propose an efficient dimensionality reduction approach that extends beyond linearity based on LD and autoencoders. Second, we provide a standardized way to tailor haploblock-specific autoencoder architectures. Lastly, we show that our strategy reduces the data to around 10% of the original size while maintaining a reconstruction accuracy exceeding 99%.

## 2 Materials and methods

### 2.1 Data description and preprocessing

We used genotyping data from Project MinE, an international effort to collect clinical and genetic data from ALS patients and healthy controls (Project MinE ALS Sequencing Consortium 2018). The data contains 23 209 ALS cases and 90 249 controls, including 56 208 males and 57 250 females from 16 European nationalities. Preceding the most extensive GWAS of ALS carried out so far, participants were gathered in cohorts according to their genotyping batch, where they passed individual and variant-level quality control (van Rheenen *et al.* 2021). Subsequently, these cohorts were merged to form five strata based on their matching genotyping platforms and were again subjected to quality control and imputation. Detailed information regarding the GWAS cohorts and five strata involved in this study can be found in Supplementary Tables S1 and S2. We reserved the participants in Stratum 1 (2254 cases and 11 155 controls) for testing, and the final results are reported on this stratum only. All our experiments leading up to the final configuration are carried out using the training dataset of 100 049 individuals (20 955 cases and 79 094 controls) formed by the remaining four strata.

We only allowed for common SNPs with minor allele frequencies (MAF) above 0.01 and excluded all SNPs with non-zero missing genotype rates, which could induce bias (Marees *et al.* 2018). As a result of these steps, 6 546 842 SNPs were kept for analysis.

To format genotype data into data that is usable by autoencoders, we encoded our SNP input to single allele dosage values of 0, 1, or 2. We resorted to *additive recoding*, i.e. allelic dosage values are obtained by counting the minor alleles at each locus per person. This conversion generates a tabular data format where each SNP is represented by a column.

All computations were executed on a system equipped with an Intel Xeon Gold 6242R processor, running at 3.10 GHz, paired with 376 GB of RAM and running Rocky Linux version 8, offered by the Utrecht Bioinformatics Center’s (UBC) high performance compute (HPC) facilities.

### 2.2 Parsing the human genome into haploblocks

Combinations of alleles that are inherited together from one parent constitute a haplotype (International HapMap Consortium 2005). The term haploblock then describes a bounded genomic region on a chromosome, harbouring only a few distinct haplotypes (Wall and Pritchard 2003). We estimated haploblock boundaries using the implemented solution (Taliun *et al.* 2014) to the Haploview algorithm (Gabriel *et al.* 2002, Barrett *et al.* 2005) in PLINK 1.9 (Purcell *et al.* 2007). Haploview delimits a haploblock over a region where only a minor share of the SNP pairs exhibit signs of historical recombination. The first step of our method is partitioning the feature space (SNPs) into these biologically defined regions, the boundaries of which should be uniform across the genomes of all included samples. SNPs contained in each haploblock constitute the input space that the associated autoencoder will not only be trained but also tested on, which is why the genomic position of a haploblock should be standardized across all samples beforehand. The authors of Haploview hypothesize that block boundaries are considerably aligned across populations, as are certain haplotypes residing in those blocks. This ensures that determining these boundaries is not too susceptible to the underlying population structure and accords with our aforementioned rationale to simultaneously consider samples from genetically diverse (European) cohorts.

To obtain lengthy, that is more crowded, blocks—advantageous for dimensionality reduction—we calibrated Haploview’s default parameters: the confidence interval for strong LD between 0.5 and 0.85, as well as the upper bound for historical recombination, which was set to 0.7 instead of 0.9. We also extended the SNP window option from 200 Kb to span 10 Mb of the genome. Such adjustments have indeed helped elongating the blocks.

### 2.3 Autoencoders

Next, autoencoders come into play to learn lower-dimensional representations of each haploblock. One by one, the models are trained to reconstruct their highly correlated SNP input, such that valuable genetic patterns can be compressed in the bottleneck.

#### 2.3.1 Model architecture and configuration

Our approach entails building haploblock-specific autoencoders. Given the large number of blocks, this process needs to be highly standardized. Due to the diversity of the number of SNPs in the blocks, it would not be ideal to impose identical architectures to each autoencoder. Conversely, designing distinct network architectures for each block would be impractical. Instead, we propose a flexible mechanism, to construct the autoencoder based on three hyperparameters, i.e. *shape*, *number of hidden layers*, and *bottleneck dimension*, which denotes the rate of compression.

Each autoencoder consists of sequential fully-connected layers. The dense connection between consecutive layers compresses the input into a lower dimension at minimum information loss, eliminating redundancy. The number of hidden layers *hl* corresponds to the number of layers between both the input and output layers and the bottleneck, hence the depths of the encoder and the decoder. The autoencoder has 2·hl + 3 layers in total.

**Figure 2.**
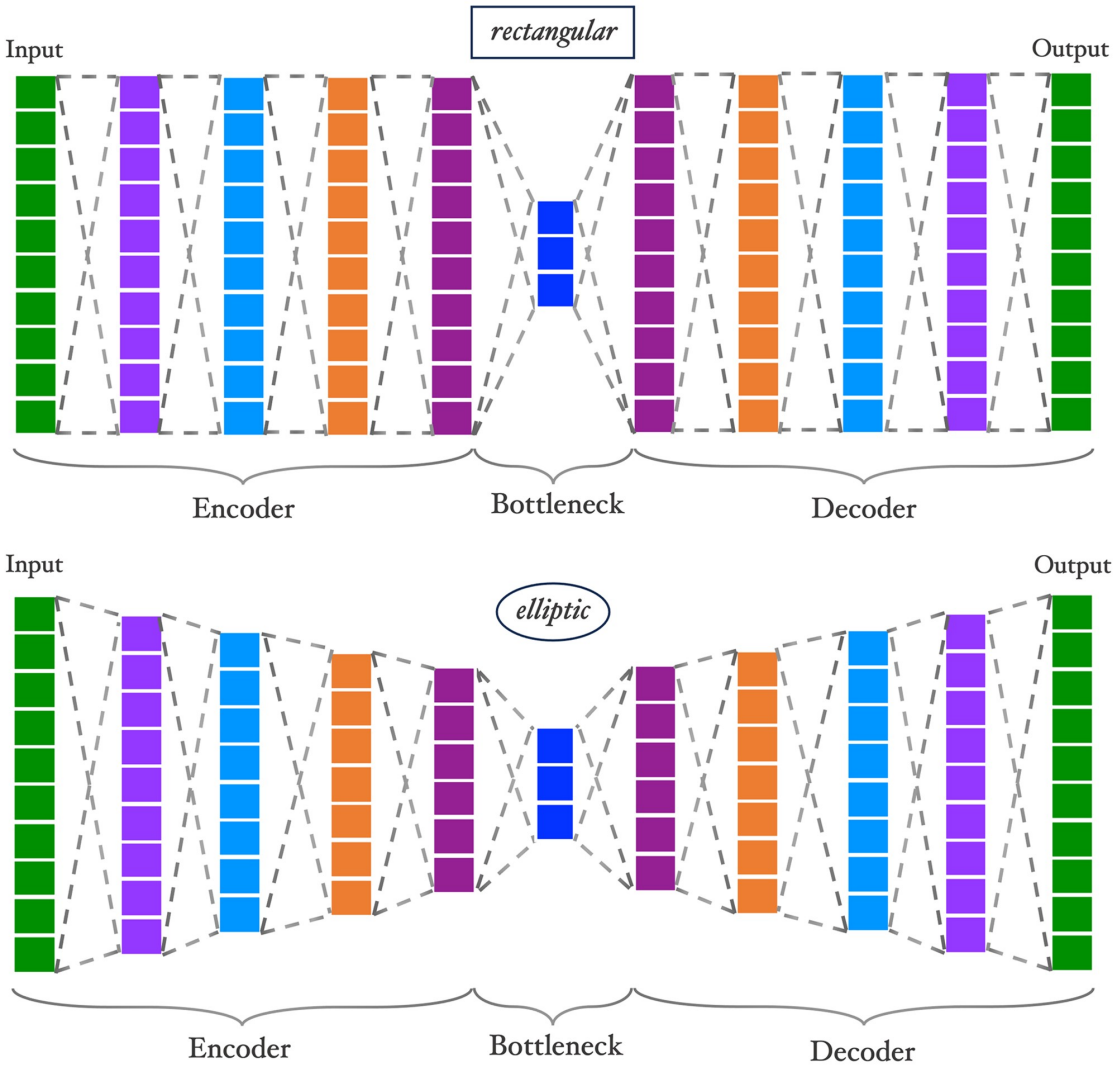
Visualizations of rectangular and elliptic autoencoders. Both networks have the same input size, number of hidden layers, and bottleneck size (inputs=10, hl=4, bn=3) and only differ by the slope *p*, see also Equation (1).

The number of neurons in each layer *l* is determined by the hyperparameter *shape*, and is computed using the bottleneck dimension (*bn*), the number of hidden layers (*hl*), and the slope of the encoder and decoder geometries (*p*). *p* can be 0 or 0.5, corresponding to shape types *rectangular* or *elliptic*, respectively (Fig. 2). The number of neurons in layer *l* is calculated as:

(1) $$n(l)=bn\;+\;\left(\frac{\text{input}\;\text{dimension}-bn}{{\left(hl\;+\;1\right)}^{p}}\cdot |l{|}^{p}\right),$$

where l∈Z, −(hl + 1) ≤ l ≤ hl + 1. The number of neurons in the input (output) layer are given by n(−(hl + 1)) (n(hl + 1)) and are equal to the input dimension. The bottleneck dimension is given by n(0) and equals *bn*. The number of neurons in a layer should always be integer, else n(l) is rounded to the nearest one.

The weights of the hidden layers are initialized with He uniform variance scaling initializer (He *et al.* 2015). We use the Leaky Rectified Linear Unit activation function (Leaky ReLU) in each layer but the output (Maas 2013). The output activations should range from 0 to 2, to reflect our recoded SNP values. Hence, we fashioned the following custom activation function, r(x), based on the tangent hyperbolic function (tan h(x)), whose output values lie between 0 and 2 instead of the original range from −1 to 1:

(2) $$r(x)=\text{tan}\;h(x)\;+\;1=\left(\frac{e^{x}-e^{-x}}{e^{x}\;+\;e^{-x}}\right)\;+\;1.$$

#### 2.3.2 Training and evaluation

We preserved 100 049 individuals (20 955 cases, 79 094 controls) in four strata (∼88.18% of data) as our training and validation subsets while the remaining stratum of size 13 409 (2254 cases, 11 155 controls) is set aside for testing. All hyperparameter tuning experiments were conducted exclusively on the training subset.

Since the SNP dosages (0, 1, 2) are of ordinal nature, we used Mean Squared Error (MSE) to quantify the reconstruction loss. Network weights are optimized using the Adam algorithm where the learning rate is initialized as 0.0001 (Kingma and Ba 2017). On each haploblock, autoencoders were trained for 50 epochs with a batch size of 32.

Two metrics are indicative of how well every single autoencoder performs: the *reconstruction loss* and the *SNP reconstruction accuracy*, hereinafter referred to as *SNP accuracy*. The former is calculated with the MSE function as a measure of the discrepancy between the input and the output values. The latter assesses the accuracy of individual predictions and is computed by rounding the predicted values of the SNPs to the nearest integer and then comparing those to the input values one by one. Thus, the SNP accuracy is the percentage of correctly predicted SNP dosages.

Moreover, we are interested in assessing to what extent the autoencoders are able to compress the haploblocks. By definition, the dimension of the bottleneck layer is equivalent to the reduced data size, and its ratio to the original input size, i.e. the number of SNPs in a haploblock, yields the compression ratio:

(3) $$\text{compression}\;\text{ratio}=\frac{bn}{\text{input}\;\text{dimension}}$$

#### 2.3.3 Hyperparameter tuning

We adopted an adjustable structure defined through three hyperparameters (*shape*, *hl*, and *bn*) which ideally need to be optimized for each haploblock individually. While tuning the hyperparameters of each model seems theoretically possible, it would require extensive memory and computation time.

In our case, the marginal gain from an extensive hyperparameter tuning procedure may not be as favorable as economizing on computational resources. To bypass the burden of tuning every autoencoder, we first selected a diverse subset of 221 haploblocks across 22 chromosomes (autosomes) and optimized hyperparameters for each of these blocks individually. To ensure that we select blocks of various lengths, in each chromosome, we grouped those in bins based on their sizes and randomly sampled one block per bin. This way, some of the blocks with a high number of member SNPs, which would have been outliers in a distribution-based selection, were represented in our subset. For each of the selected blocks, we carried out a grid search over a parameter space in which the *bn* ranges from 1 to 10, *hl* from 1 to 5, and the shape of the autoencoders is *elliptic* or *rectangular*. For this, we used 5-fold cross-validation with 100 049 training individuals to ensure that the testing stratum remained unseen by any of the trained autoencoders. Meanwhile we recorded both MSE losses and SNP accuracies of the 5-fold validation subsets for evaluation.

For downstream analyses, the bottleneck layer outputs a compressed version of the original haploblock. Choosing *bn* thus predetermines the level of dimensionality reduction. We treat *bn* as a measure for the achieved compression, as well as a hyperparameter to be optimized. Specifically, we ran the grid search for 221 haploblocks in our subset, to find the best possible combinations of *shape* and *hl* per block, under fixed *bn*. We repeated the search for every *bn* size from 1 to 10 to obtain optimal configurations while controlling for the compression level.

## 3 Results

### 3.1 Descriptive statistics of the haploblocks

Due to our method’s dependency on LD for defining haploblocks, we ruled out rare genetic variants from the original data with an MAF cutoff of 0.01. We have clustered the remaining 6 546 842 common variants into 193 122 haploblocks over 22 autosomes.

We observe in Supplementary Fig. S1 and Supplementary Table S3 that the distribution of haploblock sizes is positively skewed in each chromosome, such that most blocks contain relatively few SNPs while fewer blocks appear more populated.

### 3.2 The trade-off between compression and accuracy

**Figure 3.**
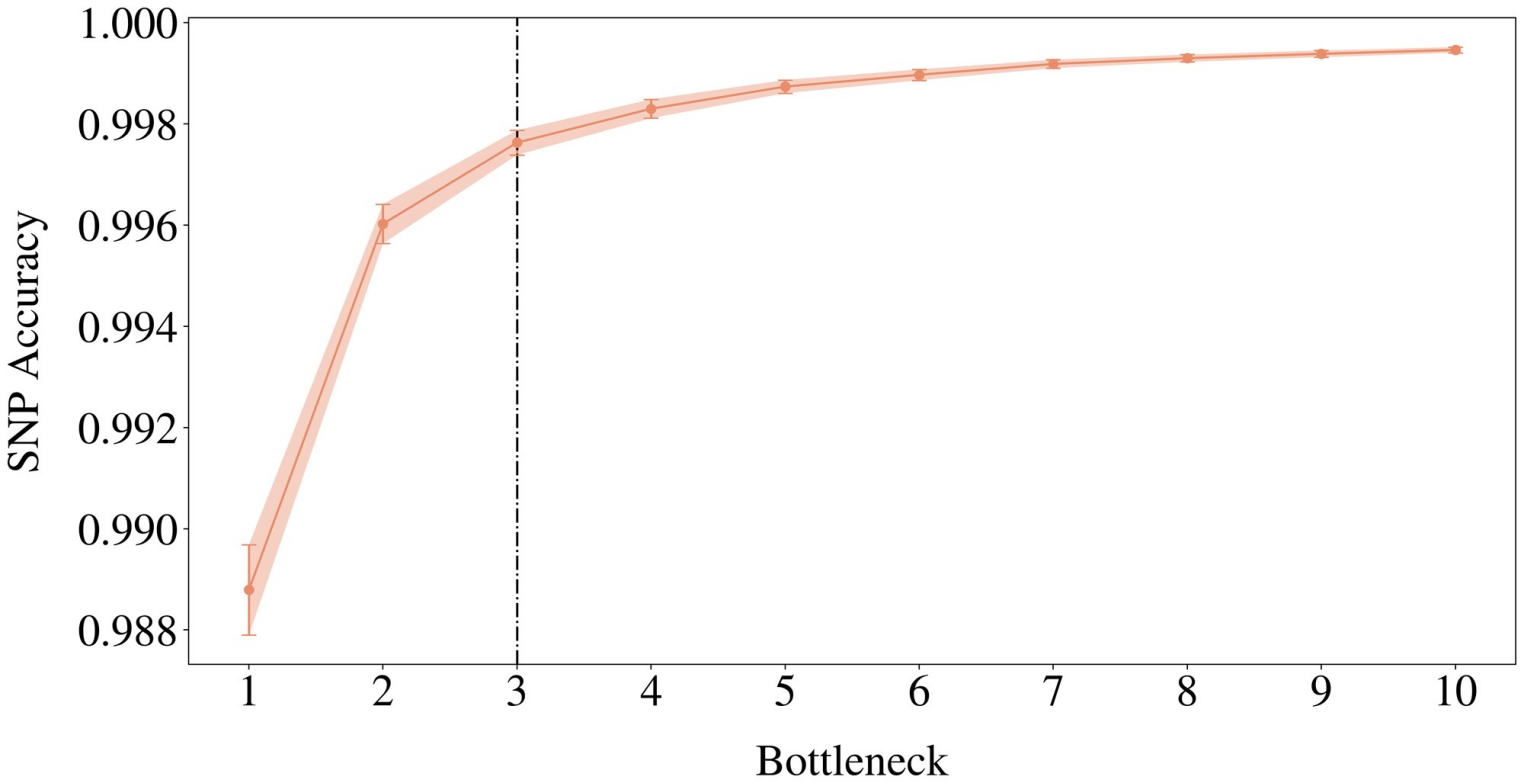
Elbow plot of validation reconstruction accuracies of the best models across *bn*∈{1,…,10}, averaged over 221 haploblocks, with 95% confidence intervals. The black line is the elbow point.

For each haploblock in our subset of 221, the grid search yielded 10 optimal hyperparameter settings corresponding to 10 values of *bn*. To examine the impact of the compressed input dimensionality on the SNP accuracy, we averaged the optimal validation accuracies over all the haploblocks for each of the 10 different *bn*. We also estimated the corresponding 95% confidence intervals, which can provide insight into the reliability of the SNP accuracy estimates across different *bn* (Fig. 3).

A wider bottleneck can lead to higher SNP accuracies by allowing for more detailed representations (Fig. 3). Also, the confidence intervals become larger on decreasing *bn*. Given the constant sample size of 221, a larger confidence interval necessarily indicates increasing standard deviation, thus a higher degree of uncertainty associated with the average SNP accuracies.

Finding the optimal bottleneck dimension involves striking a balance between a high level of compression and maintaining sufficient information for accurate reconstruction. For this, a trade-off point, where the diminishing return in SNP accuracy is no longer worth widening the bottleneck, can be found at the bottleneck size of 3 (Fig. 3). The average SNP accuracy improves only marginally (by 0.0016) with an additional bottleneck dimension between 2 and 3. Below we explore when we can prioritize compression by pushing the elbow point.

### 3.3 The influence of haploblock characteristics on accuracy

**Figure 4.**
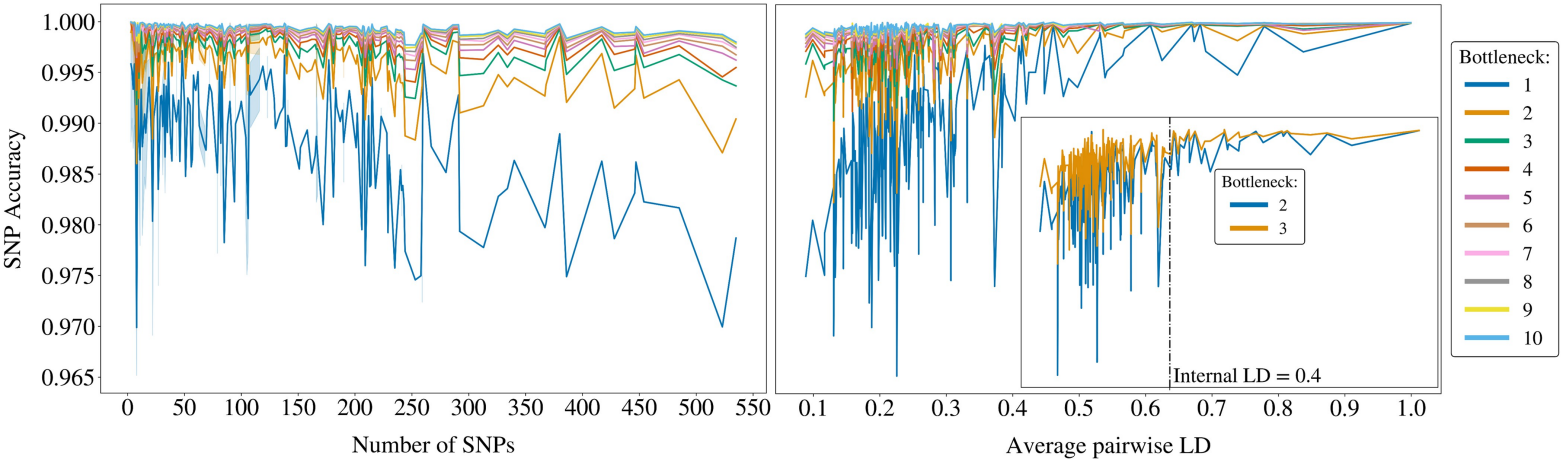
The highest validation accuracies obtained from the grid search for each block, with bottleneck values ranging from 1 to 10. The accuracies are plotted against the number of SNPs in each block (left) and against the within-block average pairwise LD (right). The zoomed-in plot on the right focuses on bottleneck values 2 and 3, offering a clearer comparison and highlighting the LD threshold at 0.4.

Every haploblock harbours a different number of SNPs. This property can be implicitly linked to the physical length of the block, hence the extent of genetic variation within (Supplementary Fig. S2). Figure 4 illustrates how the relationship between the block size and SNP accuracy is affected under different compression scales. Evidently, a more rigorous compression results in lower robustness of the SNP accuracy to increasing block sizes.

Haploblocks can otherwise be assessed by quantifying their internal genetic variation using *the average pairwise LD*. The higher the LD within a block, the more similar the haplotypes are to each other, indicating lower variation. Overall, a higher degree of compression triggers the sensitivity of SNP accuracy to the variation in blocks (Fig. 4). The positive correlation between SNP accuracy and LD gradually becomes weaker as *bn* increases.

Given the elbow at *bn *=* *3 and its minor advantage over *bn *=* *2 (Fig. 3), we zoom in to provide a clearer comparison, see Fig. 4. The SNP accuracy still seems less sensitive to changing internal LD for *bn *=* *3, although we can observe an LD threshold at 0.4, beyond which the difference between *bn *=* *2 and *bn *=* *3 becomes negligible. Below this threshold, haploblocks require more nodes in the bottleneck to ensure sufficient flow of information, as they exhibit higher internal genetic variation. Here, we can seize the opportunity to prioritize compression over accuracy by compressing the haploblocks to two dimensions, if their internal LD is above 0.4. Otherwise we stand by the original elbow at 3.

### 3.4 The cost of compression

Among the optimal hyperparameter settings for the 221 haploblocks, we counted the occurrences of each combination of *bn*, *hl*, and *shape*. The diagonal pattern with darker shades in Supplementary Fig. S3 indicates a negative correlation between *hl* and *bn*. When we track the frequencies of elliptic and rectangular architectures, a large proportion of best models has the rectangular shape for nearly all bottleneck sizes. For the majority of the haploblocks, resulting optimal models featured five hidden layers and rectangular shape when the desired bottleneck size is 2 or 3.

To gain further insight into the computational cost incurred by different levels of compression, we monitored the average time to train the best performing models across all five cross-validation folds during each block’s grid search. The average fitting times across 221 blocks decrease as the *bn* increases from 2 to 10 (Supplementary Fig. S4). Our 3-fold findings hereby suggest a need for deeper (more hidden layers) and wider (more neurons) autoencoder architectures, hence a proportional rise in computational costs, incurred by higher levels of compression.

### 3.5 Marginal returns of hyperparameter tuning

To assess the worth of a larger *hl*, we sampled all haploblocks for which the optimal *hl* is 5 and tested for a significant non-zero improvement comparing SNP accuracies resulting from different values for *hl*. Next, we conducted a similar test to compare elliptic and rectangular shapes, this time using the blocks for which rectangular is the optimal shape. Supplementary Table S4 shows results of these one-tailed *t*-tests.

The statistical analysis does not provide sufficient evidence to suggest that using *hl *=* *5 instead of *hl *=* *4 leads to a significant improvement for a *bn* of 2 and 3, at a significance level of 0.05. Yet in both bottleneck settings, the models achieve significantly higher SNP accuracies when using *hl *=* *4 compared to 1, 2, or 3. Similarly testing between rectangular and elliptic shapes resulted in *P*-values above the significance level. The return on the extra computational cost of five hidden layers or rectangular autoencoders is thus not evident. Therefore, we conclude that an elliptic autoencoder with four hidden layers can adequately compress diverse haploblocks to 2 or 3 dimensions, conditioned on the block’s average pairwise LD.

### 3.6 Compression of Chromosome 22

Using the chosen autoencoder architecture per haploblock, we compressed Chromosome 22—which originally contained 70 247 SNPs clustered in 2854 blocks—to 7341 dimensions in total. All autoencoders were trained and evaluated using the same predefined training and test samples. To assess the overall performance, we computed the average losses and accuracies along with their standard deviations across 2854 autoencoders, see Supplementary Table S5.

In the end, we achieved a dimensionality reduction down to 10.45% of the original input size with an average SNP accuracy of 99.55% on the training samples and 99.56% on the test stratum. The mean MSE losses obtained on the training and test samples were $5.69\times 10^{-3}$ and $4.75\times 10^{-3}$, respectively.

**Figure 5.**
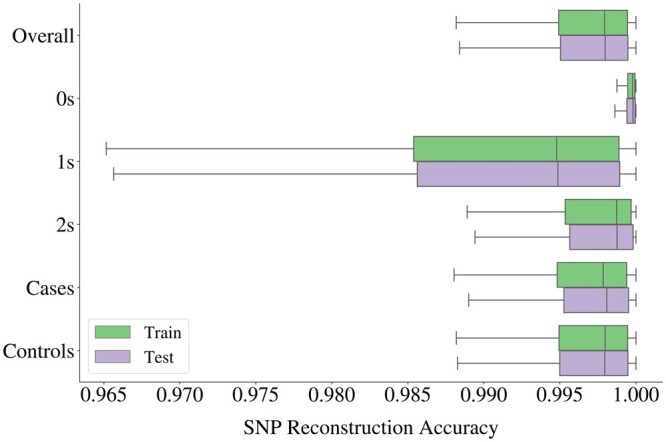
Box plot of SNP accuracies obtained from 2854 autoencoders used for compression of all haploblocks in Chromosome 22. Accuracies were also calculated for three categories of genotype dosage values (0 s, 1 s, and 2 s), and for two phenotypic groups (ALS cases and controls) as displayed on the *y*-axis.

Given the inherent sparsity of genetic input, in the sense that the most common dosage value in the data is 0, the autoencoder may simply output only 0 s and still achieve a fair SNP accuracy in highly sparse blocks. Therefore, we also evaluate the SNP accuracies of subsets in the data depicted in Fig. 5. The reconstruction of 0 s yielded the highest average accuracy, followed by 2 s, whereas the widest spread is obtained for 1 s.

Furthermore, we inspect the SNP accuracies of ALS cases and controls separately to assess whether the disease phenotype impacts the reconstruction performance. The accuracies obtained for both groups spread within highly similar ranges as can be seen from Fig. 5, and their means over all the haploblocks differ only marginally, with all values above 99.50% for both training and testing samples, see Supplementary Table S5 for details.

### 3.7 Comparison with PCA reconstruction

It is possible to reconstruct the original data from the leading principal components. For each haploblock in Chromosome 22, we applied linear transformations using PCA with the same latent space dimensionality as the autoencoder’s bottleneck layer. Here, the orthogonal basis is obtained through the same training data which is used to train the autoencoders, and the test stratum is projected to this basis only for evaluation. Subsequently, we reconstructed the original haploblocks from these principal components and calculated MSE losses and SNP accuracies by comparing the reconstructed outputs to the inputs, see Supplementary Table S6. Additionally, we calculated the *chromosome reconstruction accuracy* considering the total number of variants correctly predicted in the reconstruction across the entire Chromosome 22 as below:

(4) $$\text{chromosome}\;\text{reconstruction}\;\text{accuracy}=\frac{\sum_{i=1}^{2854}a_{i}\times b_{i}}{\sum_{i=1}^{2854}b_{i}},$$

where $a_{i}$ is the SNP accuracy of block *i* and $b_{i}$ the size of block *i*.

Job submissions were configured to utilize 32 CPU cores per task for compressing batches of eight haploblocks in parallel. Relevant computational background is provided in Supplementary Table S7.

**Figure 6.**
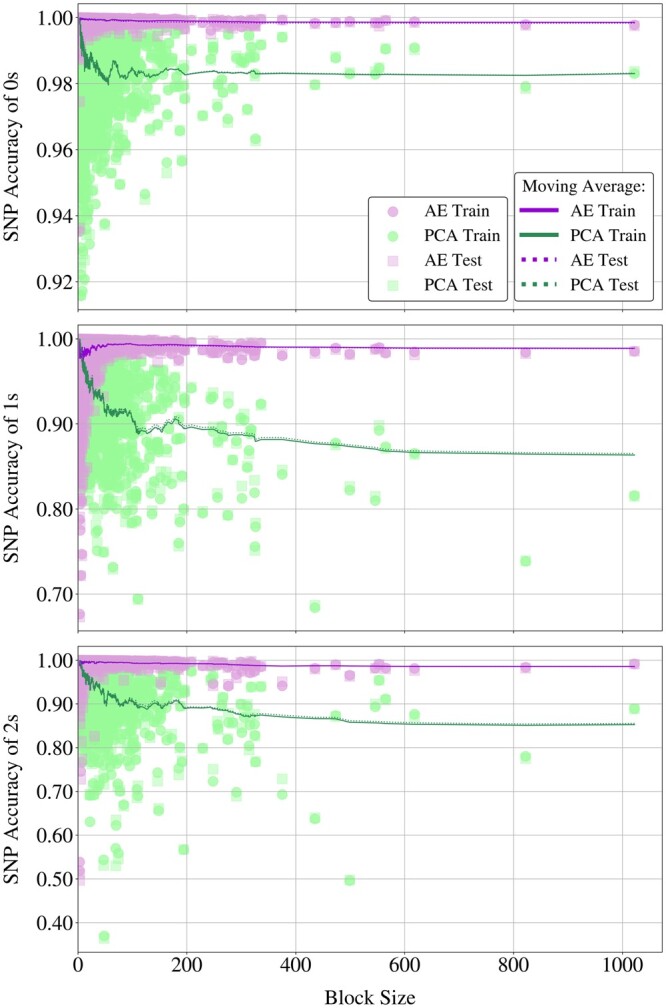
Scatter plots of SNP accuracies obtained by AE and PCA for dosage values 0, 1, and 2 (top to bottom). The *x*-axes represent block sizes, shared across plots. Solid lines illustrate the moving average of SNP accuracies on training samples using a window size of 50, dotted lines depict the same for test accuracies.

Autoencoders effectively reconstructed 99.63% of Chromosome 22 on the unseen data, while PCA achieved 96.80%, see Table 1. To pinpoint where this difference originates in, we break the SNP accuracies down based on the dosages (0, 1, or 2) and plot these against block sizes for both methods in Fig. 6.

**Table 1.** Comparison of PCA and autoencoder reconstruction through complete compression of Chromosome 22.

Method	MSE loss		Chr. Rec. accuracy	
	Train	Test	Train	Test
Autoencoder	$5.69\times 10^{-3}$	$4.75\times 10^{-3}$	**99.65%**	**99.63%**
PCA	$1.54\times 10^{-2}$	$1.48\times 10^{-2}$	96.72%	96.80%

Bold face indicates the best chromosome reconstruction accuracies.

Autoencoders consistently outperform PCA across increasing block sizes, maintaining stable performance while PCA’s accuracy declines. As given in Supplementary Fig. S5, PCA closely matches autoencoders only for the reconstruction of 1 s and 2 s in the smallest blocks with less than 10 SNPs, but the disparity in their reconstruction abilities widens evidently with increasing block sizes, see Fig. 6.

The range of the performance gap between two methods in Fig. 6 extends as dosage values become less abundant in the data. For 0 s, PCA accuracies remain above 98% and closer to autoencoders, with an erratic performance for smaller blocks. For 1 s and 2 s on the other hand, PCA accuracies diverge from the autoencoders at a much faster rate, leading to a gap that eventually exceeds 10%. Considering the sparsity of genetic data, adequate reconstruction of 0 s is necessary but insufficient to claim that the genuine SNP interactions were indeed captured. Autoencoders excel in accurately reconstructing these scarcer dosage values, also showing robustness to varying block sizes.

## 4 Discussion

This work introduced a non-linear approach to effectively compress massive genotyping data while optimizing the trade-off between retaining meaningful genetic information and low dimensionality. First, we segmented the genome into non-overlapping haploblocks that capture the LD structure of the genome. Then, we trained autoencoders to compress these haploblocks into low-dimensional representations. When these are pieced together, the resulting assembled genome becomes easier for AI approaches to digest, for example in disease prediction tasks, while allowing for explainability at the haploblock level. The prominent advantages of our approach can be attributed to several components: (i) leveraging the correlation between SNPs to facilitate compression using the haploblock arrangement, (ii) retaining the complex SNP interactions within those blocks through the layers of the autoencoder, (iii) bypassing the burdensome phase of hyperparameter tuning through a standardized autoencoder configuration, and (iv) yielding high SNP accuracies, indicating minimal loss of SNP information.

In cases where certain subpopulations are abundant in the data, autoencoders may be prone to overfitting to dominant genetic patterns driven by those, which would compromise the model’s ability to capture the genetic diversity of underrepresented groups. Namely, defining population-specific haploblock boundaries would generate population-specific feature spaces, therefore requiring training population-specific autoencoders; which might jeopardize their generalization ability. Instead, segmenting the genome using fixed boundaries enhances statistical power. The training and testing samples were both representative of the European genetic diversity carried by the rich Project MinE data, to which we owe the remarkable generalization of LD-based autoencoders to previously unseen genotypes. As previously stated, the five strata involved in this study were separated by genotyping platform. Thus, the reconstruction performances on the testing stratum are also indicative of the robustness of our approach to potential platform-specific biases, i.e. technical deviations that do not reflect the true biological variation between samples.

Imposing a fixed autoencoder architecture to each haploblock to short-circuit hyperparameter optimization would ignore the diversity of the blocks. In addition to a flexible network design with an adjustable input layer and complexity based on only three hyperparameters, our results revealed a criterion for the best configuration applicable to the entire genome. Although this generalized setting might be sub-optimal for individual blocks, it struck the balance between computational efficiency and performance. Our *compromise to optimize* strategy achieved a successful generalization of the autoencoders to an unseen stratum yielding an average test SNP accuracy of 99.63% across 2854 blocks throughout the entire Chromosome 22.

The dimensionality of each compressed haploblock can be controlled through the width of the corresponding autoencoder’s bottleneck. There is a trade-off between this width and the SNP accuracy, in other words the extent of the information retained in the low-dimensional representations of the blocks. Our methodology allows for customizing the compression settings depending on the scope of downstream tasks. For example, when predicting monogenic or maybe even polygenic traits, assuming that the causal genomic regions can be selected prior to analysis, a wider bottleneck can be chosen on account of a higher accuracy. However, for complex traits with much more intricate genetic architectures according to the hypothesis of the omnigenic model (Boyle *et al.* 2017), preferably the entire genome should be involved in the analysis. Hence, compressing more genetic information into fewer dimensions becomes the better remedy. The reconstruction performance is highly sensitive to the genetic variation (internal LD) covered by the haploblock, notably for lower bottleneck dimensions (below 4). This could mean that a wider bottleneck more steadily ensures the necessary network complexity. To provide the desired dimensionality as well as to avoid overcomplicating the networks, we considered LD-thresholding to decide between two and three bottleneck nodes.

We deduced from the grid search that the narrower the bottleneck was, the higher the complexity of the best performing models became, hence the run times of these models increased. In principle, grid search appoints the optimal setting only considering the reconstruction performance, and favors a deeper and wider network for a minor accuracy improvement, regardless of the increased training cost. The accumulated gain from economizing on run time and model complexity during each haploblock’s compression can lead to enhanced computational efficiency for the entire genome. After statistically assessing the choices made by the grid search, we seized any opportunity to simplify the networks as long as there was no statistical evidence of inferior performance.

Our findings demonstrate the performance advantage of autoencoders over PCA, which seems particularly evident in the reconstruction of 1 s and 2 s by at least 2% in mean train and test accuracies, see Supplementary Tables S5 and S6. The significance of this statistical advantage can be better grounded with reference to genotyping errors. Even when occurring at a rate below 1%, genotyping errors are deemed non-negligible as they can have serious repercussions for subsequent analyses (Pompanon *et al.* 2005, Wang 2018). Unlike 0, dosage values of 1 and 2 indicate the presence of a minor allele at a particular genetic locus, hence existence of a SNP. As a linear transformation, PCA’s limited ability to capture these values hints at the intricacy and non-linearity of the SNP interactions within a block. Despite its low maintenance and cost-effectiveness, breaking the non-linearity in the process and the associated risk of losing epistasis renders PCA unappealing for compressing the genome, an issue that can be overcome with autoencoders.

Although our approach is promising for developing AI-applicable representations of haploblocks, there are potential areas for improvement. First, the foundation of our method is to segment the genome using the LD structure, not directly applicable to the rare variants (Zhang *et al.* 2002), which were excluded from the data beforehand. Hence the haploblocks in this work contain only the common SNPs (MAF above 0.01) by definition. Besides, even if rare SNPs could be considered in the analysis, the latent space learnt by the autoencoders might not authentically represent the rare variation due to the low frequency of such variants in the population. However, regarding ALS in particular, discovering the pathogenicity of rare variants could play a major role in resolving the genetic mystery of the disease (van Rheenen *et al.* 2021). This renders the further development of compression or feature learning strategies focused on rare variants inevitable. Also, estimating the boundaries of haploblocks involves presetting a scanning window for SNPs (10 Mb in this study). Therefore, the long-range dependencies between physically distant genetic variants are not necessarily covered by the haploblocks and they might not be captured throughout compression. Fortunately, the corpus for modeling the long-range dependencies in sequential data is progressing fast and showing encouraging outcomes in genomics (Ji *et al.* 2021, Nguyen *et al.* 2024) and our compressed representations form suitable inputs for modeling such dependencies at the haploblock level. Lastly, our final results display the effectiveness of our method only on Chromosome 22, as a practical demonstration. Here, it should be acknowledged that the LD structure varies between different chromosomes (De La Vega *et al.* 2005), leading to potential differences in haploblock characteristics; therefore the outcomes of our method on Chromosome 22 may not invariably generalize to Chromosomes 1–21. In one respect, if a chromosome holds larger haploblocks with lower variation within, our system could prove more effective in terms of reduced dimensionality and reconstruction accuracy, and vice versa.

## 5 Conclusions and future work

In this article, we presented a dimensionality reduction approach that makes massive genome data compatible with AI techniques. To retain local patterns, we first partitioned the genome into clusters of correlated SNPs, forming haploblocks. Then, we trained autoencoders capable of capturing intricate relationships to compress each block. Given the unique characteristics of these blocks, we devised adjustable autoencoder architectures using only three hyperparameters. Our findings revealed a criterion for the optimal hyperparameters, which helped mitigate their resource-intensive tuning process. Evaluation of our method on an entire chromosome showcased the unprecedented potential of leveraging the LD structure of the genome in conjunction with autoencoders for data compression, with notably high reconstruction performances. Such a synergy between LD and autoencoders holds promise for effectively preprocessing the genome, eventually to demystify complex disease genetics.

Our compression approach enables various possibilities, including an alternative to single SNP-association tests. The compressed representations can be utilized for conducting haploblock association studies. While the former might offer higher resolution at the SNP level, the latter can provide additional insights into the genetic architecture of complex traits by capturing the effects of multiple closely linked SNPs (Hirschhorn and Daly 2005, Hayes 2013). Furthermore, drawing inspiration from the denoising autoencoders (Vincent *et al.* 2010), which reconstruct noiseless targets from perturbed inputs, our method holds significant potential for reference-free missing SNP imputation. Lastly, the self-supervised nature of autoencoders can be modified by conditioning their learning through a supervised task (Dincer *et al.* 2020). We plan to expand our approach with an auxiliary ALS classification task, pursuing the missing heritability of the disease.

## Supplementary Material

btae326_Supplementary_Data
